# Focal nodular hyperplasia: hepatobiliary enhancement patterns on gadoxetic-acid contrast-enhanced MRI

**DOI:** 10.1007/s00261-012-9916-0

**Published:** 2012-06-24

**Authors:** C. S. van Kessel, E. de Boer, F. J. W. ten Kate, L. A. A. Brosens, W. B. Veldhuis, M. S. van Leeuwen

**Affiliations:** 1 Department of Radiology, University Medical Center Utrecht, Heidelberglaan 100, 3584 CX Utrecht, The Netherlands; 2 Department of Surgery, University Medical Center Utrecht, Heidelberglaan 100, 3584 CX Utrecht, The Netherlands; 3 Department of Pathology, University Medical Center Utrecht, Heidelberglaan 100, 3584 CX Utrecht, The Netherlands

**Keywords:** Focal nodular hyperplasia, Gadoxetic acid, Magnetic resonance imaging, Histology, Enhancement characteristics

## Abstract

**Objectives:**

To assess the range of hepatobiliary enhancement patterns of focal nodular hyperplasia (FNH) after gadoxetic-acid injection, and to correlate these patterns to specific histological features.

**Materials and methods:**

FNH lesions, imaged with Gadoxetic-acid-enhanced MRI, with either typical imaging findings on T1, T2 and dynamic-enhanced sequences or histologically proven, were evaluated for hepatobiliary enhancement patterns and categorized as homogeneously hyperintense, inhomogeneously hyperintense, iso-intense, or hypo-intense-with-ring. Available histological specimens of FNHs (surgical resection or histological biopsy), were re-evaluated to correlate histological features with observed enhancement patterns.

**Results:**

26 FNHs in 20 patients were included; histology was available in six lesions (four resections, two biopsies). The following distribution of enhancement patterns was observed: 10/26 homogeneously hyperintense, 4/26 inhomogeneously hyperintense, 5/26 iso-intense, 6/26 hypointense-with-ring, and 1/26 hypointense, but without enhancing ring. The following histological features associated with gadoxetic-acid uptake were identified: number and type of bile-ducts (pre-existent bile-ducts, proliferation, and metaplasia), extent of fibrosis, the presence of inflammation and extent of vascular proliferation.

**Conclusion:**

FNH lesions can be categorized into different hepatobiliary enhancement patterns on Gadoxetic-acid-enhanced MRI, which appear to be associated with histological differences in number and type of bile-ducts, and varying the presence of fibrous tissue, inflammation, and vascularization.

Focal nodular hyperplasia (FNH) is the second most frequent benign liver lesion after hemangioma and it is the most common solid benign liver lesion, comprising ~8 % of all primary hepatic tumors [1–4]. FNH is a well-circumscribed, usually solitary mass, characterized by a central fibrous scar with surrounding nodules of hyperplastic hepatocytes and small bile ductules. No normal portal structures are noted, although major vessels may course through the tumor and are prominent in the fibrous scar [2, 5]. FNH is considered to be the result of a vascular malformation, which leads to hepatocellular hyperplasia [5, 6]. Considering the bile-ducts within FNH, Butron et al. [7, 8] have proposed that the ductular component is not only a proliferation of pre-existing bile-ducts, but also the result of hepatocellular ductular metaplasia. FNH’s do not have the potential for malignant transformation and they are not known to cause bleedings like adenoma [9]. Clinical symptoms due to mass effect are infrequent. For these reasons, a lesion diagnosed as FNH in general does not require any treatment or follow-up.

Owing to the increased use of cross-sectional imaging techniques, the incidental finding of a solid liver lesion in a patient without chronic liver disease or a known primary tumor is a frequent occurrence in daily radiological practice. In case of such an incidental finding, or incidentaloma, it is important to differentiate between FNH, which does not require any treatment or follow-up, from other solid liver lesions like adenoma, hepatocellular carcinoma, or metastases, as these lesions require either surveillance or medical or surgical treatment [9].

For the diagnosis of FNH, contrast-enhanced CT, contrast-enhanced MRI, or contrast-enhanced ultrasound (CEUS) is frequently performed. Previous studies have identified typical radiological features of FNH’s for these imaging modalities [10–17]. However, many studies have reported atypical appearances of FNH on CT, MRI or CEUS, which may hamper the individual diagnostic process. The incidence of atypical findings varies widely between these reports (10–80 %) [18–21]. Consequently, lesions with atypical findings frequently require further diagnostic work-up or follow-up, which is time-consuming, costly and creates unrest for the patient.

Recently, Gadoxetic acid (Primovist®, Bayer Schering Pharma, Berlin, Germany) has been introduced as a liver-specific contrast agent which may facilitate the differentiation between FNH and non-FNH lesions. Gadoxetic acid is a hepatobiliary contrast agent which allows dynamic arterial and portal phase imaging, followed by a hepatobiliary phase at 10–20 min. It is equally cleared by the kidneys and the liver. During the hepatobiliary phase, progressive contrast uptake is observed in normal functioning liver parenchyma, followed by enhancement of the bile-duct system, as the contrast is transported to the biliary canaliculi and subsequently to the extrahepatic bile-ducts [22]. Zech et al. [17] reported contrast enhancement during the hepatobiliary phase in ~90 % of FNH’s; as opposed to other focal liver lesions, such as metastases, hepatocellular carcinoma or adenoma, which generally do not show enhancement during the hepatobiliary phase. However, 5–10 % of HCC lesions are reported to demonstrate hepatobiliary contrast uptake, possible related to OATP-1B1 and/or -1B3 gene-expression and the presence of bile-duct elements [23]. Zech et al. [17] reported that the majority of FNH lesions are either homogeneously or inhomogeneously hyperintense in the hepatobiliary phase, while a minority demonstrates no enhancement or only peripheral enhancement. No explanation as to the actual mechanism underlying the different enhancement patterns was postulated.

This study was designed to meet two goals: 1. to assess the range of hepatobiliary enhancement patterns of FNH lesions after administration of gadoxetic acid, and 2. to correlate the hepatobiliary enhancement patterns of FNH lesions to specific histological features in available histological specimens obtained after resection or biopsy.

## Patients and methods

### Identification of FNH lesions

This study was Institutional Review Board approved. Informed consent was waived as no additional investigation was required and the study was performed using data from routine clinical care.

Between January 2007 and May 2011, 133 consecutive patients underwent Gadoxetic acid-enhanced MRI of the liver, according to the MR imaging protocol summarized in Table 1. Of these, 50 patients had colorectal liver metastases, and 21 patients were known to have chronic liver disease with known or suspected HCC. Another 62 patients underwent Gadoxetic acid-enhanced MRI scan of the liver for characterization of a focal liver lesion, detected as an incidental finding during previous imaging. In this study, we will focus on this latter group of patients.

**Table 1:** MR imaging protocol

Pulse sequence	Plane	TR	TE	Flip	FOV (mm)	Gap (mm)	Slice (mm)	Matrix
SURVEY insp	Axial	2.5	1.27	50	450	3.5	8	192 × 144
SURVEY exp	Axial	2.5	1.27	50	450	3.5	8	192 × 144
Refscan	Axial	8.0	0.57					56 × 40
T1 TFE bh, insp	Axial	8.5	4.2	10	450	0	10	256 × 128
T1 TFE bh, insp	Sagittal	8.5	4.2	10	450	0	10	256 × 128
T1 TFE bh, insp	Coronal	8.5	4.2	10	450	0	10	256 × 128
T1 TFE bh in + out of phase	Axial	181	2.3/4.6	80	375	1	7	224 × 134
T1 FFE RT	Axial	10	4.6	15	405	1	7	256 × 126
T1 THRIVE bh (Pre-contrast, 25 and 60 s, 3, 5, and 10 min)^a^	Axial	3.7	1.76	10	450	−2	4	176 × 124
T2 TSE RT	Axial	556	80	90	405	1	7	400 × 215
EPI-DWI *b* = 0, 50 fb, RT	Axial	4095	56	85	360	0	5	128 × 83
EPI-DWI *b* = 0, 500 fb, RT	Axial	4095	56	85	360	0	5	128 × 83
THRIVE bh 20 min^a^	Axial	3.7	1.76	10	450	−2	4	176 × 124

*TR* repetition time; *TE* echo time; *flip* flip angle; *FOV* field of view; *slice* slice thickness; *TFE* turbo field echo; *TSE* turbo spin echo; *FFE* fast field echo; *EPI* echo planar imaging; *SSH* single shot; *RT* respiratory triggered; *bh* breath hold; *fb* free breathe; *THRIVE* T1 weighted high resolution isotropic volume examination

MRI scans were performed on a 1.5 Tesla MRI scanner (Philips, Best, The Netherlands) using a SenseBody coil

MRI scans were stored in the Picture Archiving and Communication System at the UMC Utrecht (PACS image viewer, Easy Vision Workstation, Philips Medical Systems, The Netherlands

^a^ After injection of Gd-EOB-DTPA 0.25 μmol/kg bolus at 2 mL/s through an intravenous cubital line, followed by a 25 mL saline chaser

To identify FNH lesions, all 62 MRI exams were reviewed during a consensus-reading with a hepatobiliary radiologist (MvL 25 years experience), a radiologist specializing in abdominal radiology (EB 6 years experience) and a research fellow (CvK). First, non-contrast-enhanced T1 and T2 weighted sequences, and early dynamic sequences (25, and 60 s) were reviewed in order to assess lesion appearance during the different sequences. A six-point scale was used to describe lesion intensity as compared to surrounding liver parenchyma: 1—markedly hypo-intense, 2—moderately hypo-intense, 3—iso-intense, but visible due to mass effect, 4—moderately hyperintense, 5—markedly hyperintense, 6—not visible (iso-intense without mass effect, but visible on at least one other sequence). If a scar was present, the same six-point scale was used to describe the signal intensity of the scar compared to the surrounding lesion. Secondly, stringent criteria, described in prior studies to be pathognomonic for FNH, were applied to each detected liver lesion (Table 2).

**Table 2:** Overview of typical and atypical FNH features

	Typical features	Atypical features
Lesion	Lesion enhancement is homogeneous during all phases.	Marked lesion heterogeneity
T1	Moderately hypo- or iso-intense	Strongly hypo- or hyper-intense
T2	Moderately hyper- or iso-intense	Strongly hyper- or hypo-intense
Arterial	Intense arterial enhancement	Minimal or no enhancement
Portal-venous	Hyperintense (rapid loss of signal intensity compared to arterial phase) or iso-intense	Hypo-intense
Equilibrium	Iso-intense or moderately hyperintense	Hypo-intense or strongly hyper-intense
Scar	Obligatory for typical FNH if lesion is >3 cm, and may be present if lesion is <3 cm	Absence of scar in lesions >3 cm
	Presents as a linear or stellate area in the centre of the lesion.	
T1	Hypo-intense, relative to the surrounding lesion	Hyper-intense
T2	Hyper-intense, relative to the surrounding lesion	Hypo-intense
Arterial	Non-enhancing	
Portal-venous	Hypo-intense, relative to the surrounding lesion	
Equilibrium	Moderately hyperintense or iso-intense	Hypo-intense

FNH lesions included for further study were either (a) typical FNH lesions on imaging, demonstrating all typical FNH features described in Table 2, and requiring no further treatment or follow-up as decided by the weekly convening multidisciplinary tumorboard; or (b) lesions with one or more atypical findings on imaging, that were only characterized as FNH after histological examination of the resected specimen or histological biopsy.

### Enhancement patterns in hepatobiliary phase

Subsequently the hepatobiliary phases (5 and 10 post-contrast injection) of all FNH lesions were evaluated in order to identify their hepatobiliary enhancement patterns. Signal intensity in the hepatobiliary phase was assessed using the above described six-point scale, varying from markedly hypo-intense to markedly hyper-intense relative to the surrounding liver parenchyma. In addition, based on prior observations, enhancement patterns were categorized into one of the following four patterns: (1) homogeneously hyperintense pattern: homogeneously hyperintense signal intensity in all parts of the lesion with possible exception of a central stellate or linear hypodensity; (2) inhomogeneously hyperintense pattern: hyperintense signal intensity of the lesion with scattered 1–5 mm areas of hypo-intense signal intensity throughout the lesion; (3) iso-intense pattern: lesion is visible due to mass effect, but with iso-intense signal intensity, and finally: (4) “hypo-intense-with-ring” pattern: predominant, non-enhancing, spherical centre surrounded by a thin peripheral rim of hyper-intense signal intensity.

### Histological examination

The available histological specimens of FNH, either from surgical resection or histological biopsy, were re-evaluated in order to correlate the histological features with the observed enhancement patterns. A dedicated pathologist with hepatobiliary experience (FK 35 years experience) reviewed all specimens in order to identify and localize bile-ducts and to characterize bile-ducts into one of three types, i.e., (1) pre-existent bile-ducts; (2) bile-duct proliferation (rather well-differentiated bile-ducts proliferating secondary to increased pressure); or (3) bile-duct metaplasia (dedifferentiation, or metaplasia of hepatocytes into non-functioning bile-ducts). Furthermore, the pathologist assessed the presence of inflammation and abnormal vascular configurations. For the characterization of the bile-ducts immunohistochemical analysis with CK7 and CK19 was performed (see Table 3). Immunohistochemical analysis with CD34 was performed to assess the intratumoral vasculature and sinusoidal vessels. In addition, Azan staining was performed to demonstrate the extent of fibrous tissue.

**Table 3:** Overview of CK7, CK19 and CD34 characteristics

Glycoprotein	Function
CK 7	Cytokeratin 7 is a protein belonging to the type I Keratin family, and is used to identify bile-ducts. The keratins are intermediate filament proteins responsible for the structural integrity of epithelial cells. CK 7 in hepatocytes is upregulated by cholestasis and therefore an upregulation of CK 7 is seen in areas with dedifferentiation of normal hepatocytes to bile-ducts (ductular metaplasia) and areas with bile-duct proliferation
CK 19	Cytokeratin 19 is also a protein belonging to the type I Keratin family, and is also called a bile-duct type keratin. In the liver CK 19 is expressed by well-differentiated (native) bile-ducts and can therefore be present in focal nodular hyperplasia. CK 19 expression can therefore be observed in areas with pre-existent bile-ducts and ductular proliferation
CD34	CD 34 is a cell surface glycoprotein that functions as cell–cell adhesion factor. CD 34 is expressed by (premature) hematopoietic and vascular associated tissue. CD 34 expression by sinusoid endothelial cells is associated to the process of angiogenesis. Diffuse expression of CD 34 is a sign of carcinogenesis and is seen in hepatocellular carcinoma. Non-diffuse expression of CD34 can be seen in FNH in areas with sinusoidal proliferation

## Results

### Identification of FNH lesions

**Fig. 1:**
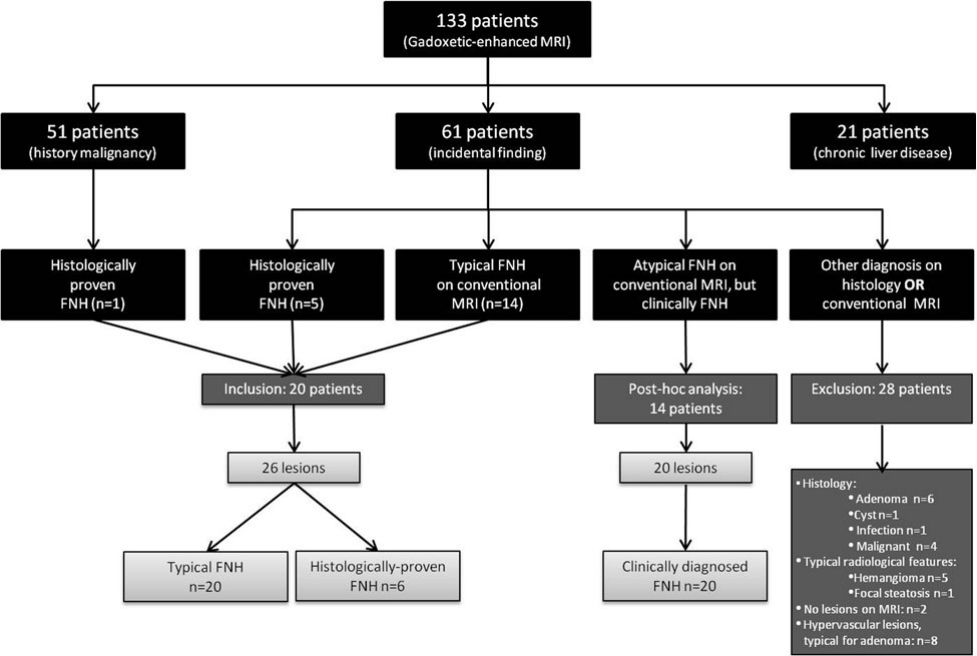
Flowchart of FNH identification

On pre-contrast and early dynamic MRI, a total of 21 typical FNH’s were identified in 15 patients. Although presenting with a typical appearance on MRI, histopathology was acquired in one of these lesions. This lesion occurred in a patient referred with a presumed colorectal liver metastasis and for that reason surgical resection was performed. Histopathology was available in another 5 FNH’s in 5 patients (3 surgically treated, 2 histological biopsies), in whom the MRI appearance and enhancement pattern were not pathognomonic for FNH. This resulted in a total of 26 FNH’s, varying in size between 0.5 and 11.9 cm (mean 3.1 cm) in 20 patients (15 women, 5 men; mean age 46.1 years, range 28.8–60.9 years). 15 lesions were less than or equal to 3 cm in diameter and 11 lesions were larger than 3 cm in diameter. Four patients presented with more than one FNH (range 1–4), the other 16 patients presented with a solitary lesion. For further details see Fig. 1.

### FNH appearance on hepatobiliary images

**Fig. 2:**
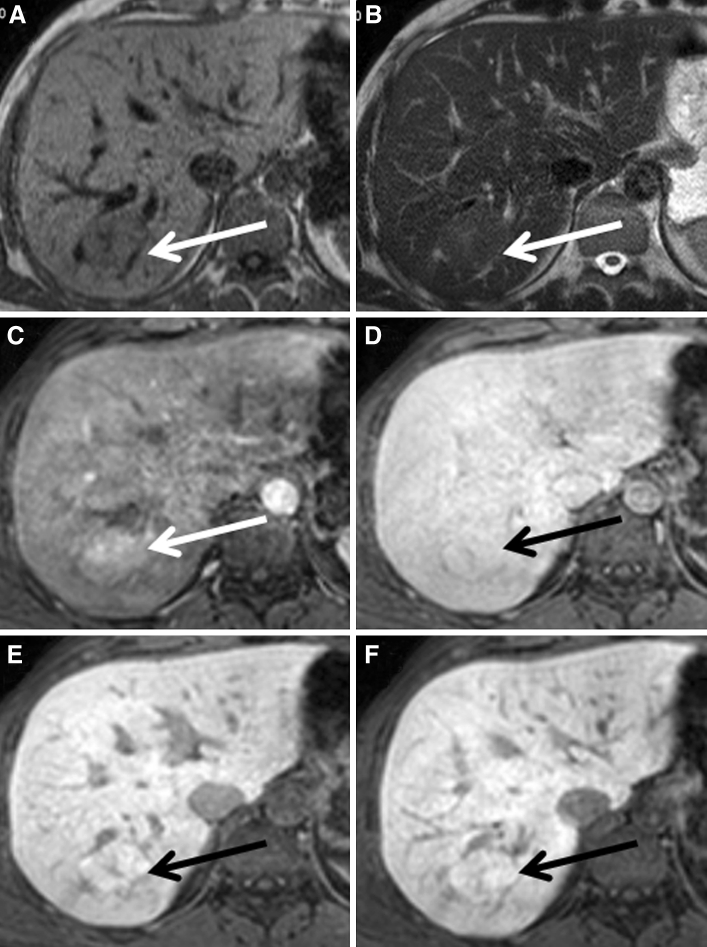
A patient presenting with a typical FNH on conventional FNH, which appeared homogeneously hyperintense to the surrounding parenchyma during hepatobiliary phases. **A**–**F** The FNH is visible on T1, T2, and during arterial phase, portal-venous phase, and 5 and 10 min hepatobiliary phase, respectively (*white* and *black* *arrows*).

**Fig. 3:**
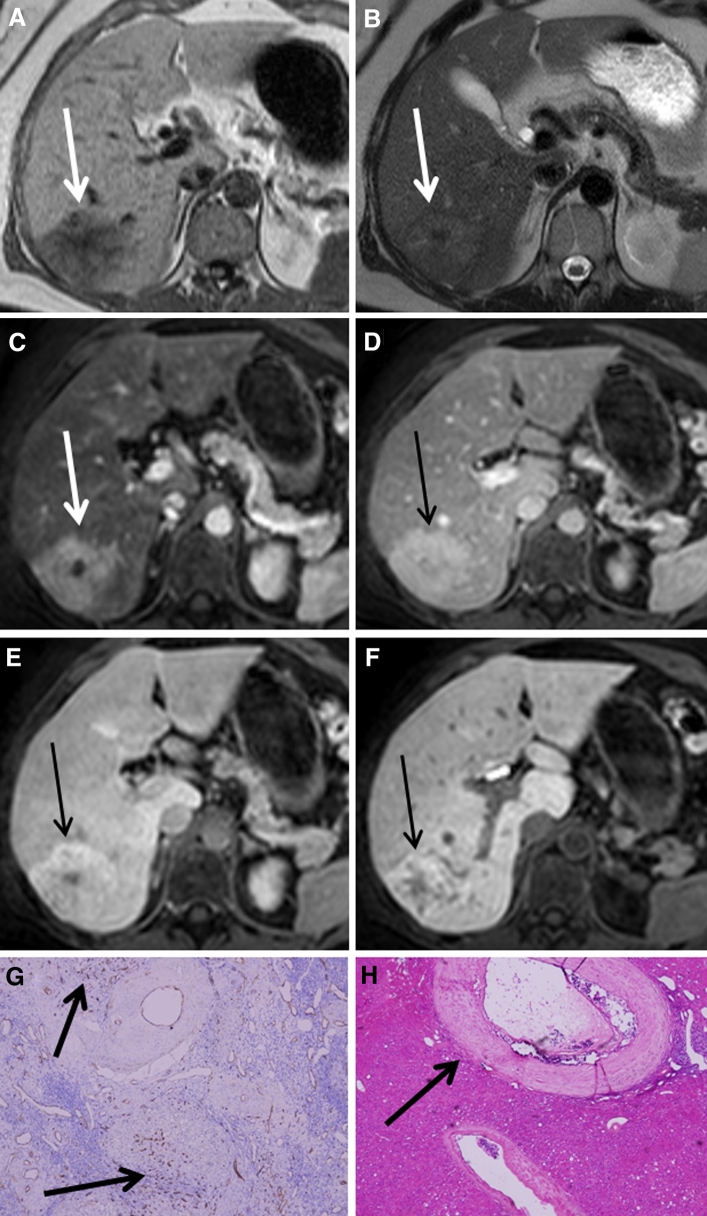
A patient with a histologically proven FNH presenting as an inhomogenously hyperintense lesion on hepatobiliary phases (Tumor C). **A**–**F** The FNH is visible on T1, T2, and during arterial phase, portal-venous phase, and 5 and 10 min hepatobiliary phase, respectively (*white* and *black arrows*). Initially, the lesion presents as a hyperintense lesion with a non-enhancing central scar during arterial phase, but over time the lesion characteristics change resulting in an inhomogeneous appearance during hepatobiliary phase. **G** CD34 immunohistochemistry shows diffuse positivity throughout the lesion (*brown* staining at *arrows*). **H** Hematoxylin and eosin staining showing an abnormal, enlarged vessel with thickened vessel wall (*arrow*). The inhomogeneous appearance on hepatobiliary phase is presumably related to areas with ischemic injury and ductular metaplasia due to vascular abnormalities, which are alternated by areas with ductular proliferation.

**Fig. 4:**
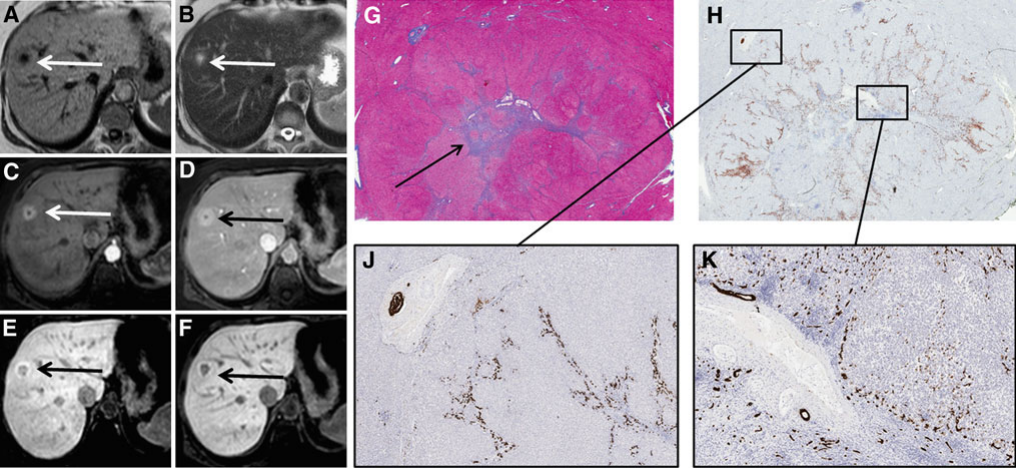
Patient with a histologically proven FNH (Tumor A) presenting as a hyperintense lesion during early dynamic phases and presenting as a hypo-intense-with-ring type FNH during hepatobiliary phases. **A**–**F** The FNH is visible on T1, T2, and during arterial phase, portal-venous phase, and 5 and 10 min hepatobiliary phase, respectively (*white* and *black arrows*). The FNH is visible during all phases and initially presents as a hypervascular lesion on arterial phase with a central scar. In hepatobiliary phase, a larger hypo-intense core develops, whilst the periphery of the lesion is persistently hyperintense. **G** Azan staining on a whole mount section of the lesion with the presence of abundant fibrous tissue in the center (arrow). **H** CK7 immunohistochemistry on the resection specimen (whole mount). *Brown* colored areas represent ductular proliferation and ductular metaplasia. **J** an enlargement of the periphery of the lesion showing pre-existent bile-ducts, ductular metaplasia and some ductular proliferation, while **K** is an enlargement of the lesion centre surrounding the fibrous tissue, showing a combination of ductular proliferation and ductular metaplasia, although the ductular metaplasia is more pronounced.

**Fig. 5:**
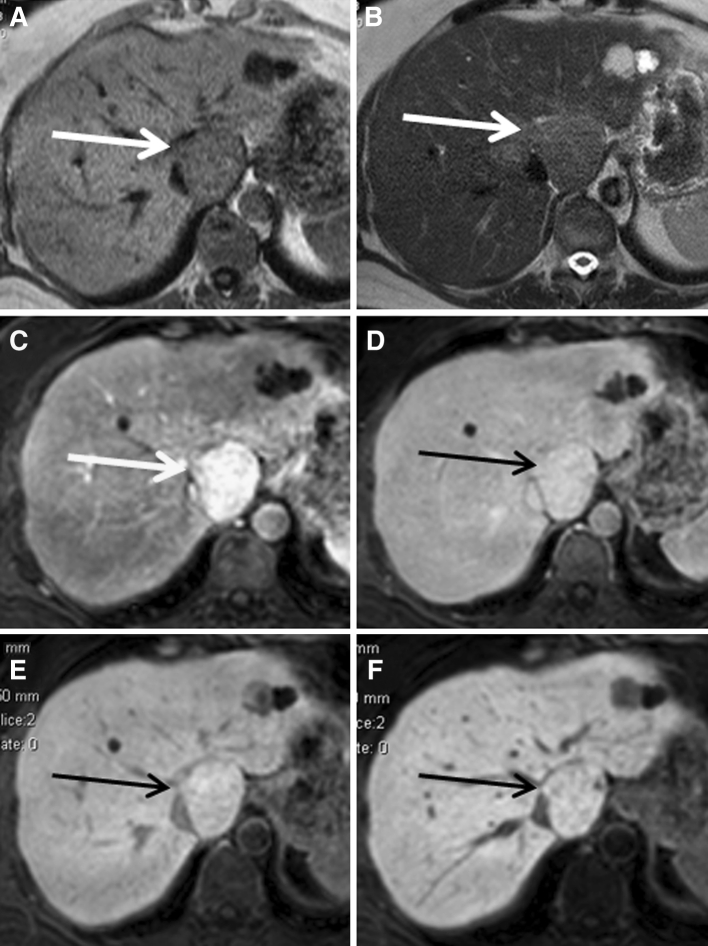
A patient presenting with a typical FNH on conventional FNH, which appeared iso-intense to the surrounding parenchyma during hepatobiliary phases. **A**–**F** The FNH is visible on T1, T2, and during arterial phase, portal-venous phase, and 5 and 10 min hepatobiliary phase, respectively (*white* and *black arrows*).

Ten lesions (10/26, 38 %) demonstrated a homogeneously hyperintense pattern (mean size 2.3 cm, range 0.5–6.4 cm; 3 lesions >3 cm, 7 lesions <3 cm) (Fig. 2a–f). A thin, stellar or linear hypodensity, interpreted as the central scar on early dynamic sequences, was visible during the hepatobiliary phases in all 3 lesions larger than 3 cm and in none of the lesions smaller than 3 cm. Four lesions (4/26, 15 %) showed an inhomogeneously hyperintense pattern (Fig. 3a–f), where 1–5 mm nodular areas of hypo-intense signal were scattered throughout the enhancing lesion without the presence of a typical scar (mean size 5.9, range 2.5–11.9 cm, one lesion <3 cm, 3 lesions >3 cm). Six lesions (6/26, 23 %) demonstrated a “hypointense-with-ring” pattern (mean size 1.9 cm, range 1.0–2.6 cm, all lesions <3 cm) (Fig. 4a–f). Five lesions (5/26, 19 %) showed an iso-intense pattern with mass effect (mean size 3.5 cm, range 1.7–5.3 cm; 2 lesions <3 cm, 3 lesions >3 cm) (Fig. 5a–f), with a scar present in all three lesions larger than 3 cm.

**Fig. 6:**
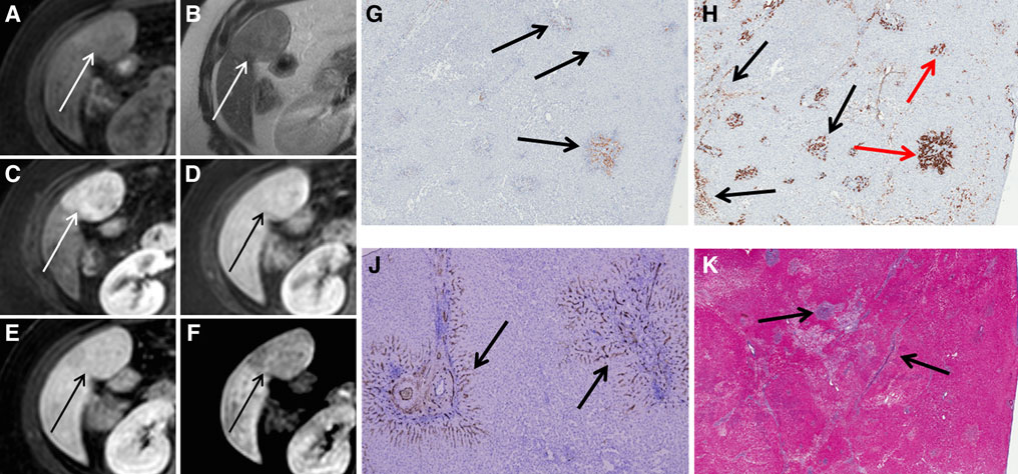
Another patient presenting with a hypo-intense type FNH in segment 5 (Tumor B). **A**–**F** The FNH is visible on T1, T2, and during arterial phase, portal-venous phase, and 5 and 10 min hepatobiliary phase, respectively (*white* and *black arrows*). The FNH is visible during all phases and initially presents as a hypervascular lesion on arterial phase without a central scar. During hepatobiliary phases, the lesion signal is less intense compared to the surrounding parenchyma, resulting in a slightly hypointense aspect. **G** CK 19 immunohistochemistry showing weakly CK 19 positive ductular proliferation in the lesion periphery (*arrows*), while the lesion centre is almost completely negative. **H** CK7 immunohistochemistry shows diffuse positivity in the lesion representing both ductular proliferation and ductular metaplasia (*black arrows*); the bile-ducts that are positive for CK19 (**G**) are positive for CK7 as well (*red arrows*). The ductular metaplasia is more pronounced than the ductular proliferation and is visible both in the lesion centre as in the lesion periphery. **J** CD34 immunohistochemistry shows expression around the fibrous tissue (*arrows*). **K** Azan staining demonstrates the presence of fibrous tissue throughout the lesion (*black arrows* pointing out *blue areas*), although a central scar cannot be identified.

One lesion could not be categorized into one of these four patterns. This lesion (5.5 cm) appeared hyperintense during early dynamic phases, but signal intensity decreased during hepatobiliary phases, resulting in a slightly hypo-intense signal compared to the surrounding parenchyma. This lesion was homogeneously hypo-intense, and did not show a peripheral rim like the “hypo-intense-with-ring” type FNH’s (Fig. 6a–f).

### Correlation between histopathological features and appearance on hepatobiliary phase

Histopathology was available in six lesions (further referenced as Tumors A–F); four resection specimens (Tumors A–D) and two core-biopsies (Tumors E and F) (see Table 4).

**Table 4:** Evaluation of six histologically proven FNH’s, Tumor A–F, respectively

	Tumor A	Tumor B	Tumor C	Tumor D	Tumor E	Tumor F
CK 19	Moderately positive: only lesion periphery shows a limited number of pre-existent bile-ducts and some ductular proliferation	Positive: lesion periphery shows stronger CK19 positivity due to ductular proliferation but no pre-existent bile-ducts. Lesion centre is almost completely negative for CK 19	Positive: areas with ductular proliferation of CK19 positive bile-ducts and pre-existent bile-ducts	No expression: only a few normal pre-existent bile-ducts, while normal parenchyma is strongly positive	Biopsy → insufficient tissue to perform CK19 staining	Positive: both pre-existent bile-ducts and bile-duct proliferation
CK 7	Markedly positive: mostly ductular proliferation around the tendrils of the fibrous scar, less ductular metaplasia	Strongly positive (++): in lesion centre mainly due to ductular metaplasia and only limited due to ductular proliferation. Lesion periphery also reveals combination of ductular metaplasia and proliferation	Strongly positive (++): especially the areas with ductular metaplasia and dedifferentiated hepatocytes due to bile flow obstruction show strong CK7 positivity. Little ductular proliferation	Strongly positive (++): positive throughout lesion, caused by well-differentiated ductular proliferation. There are no signs of metaplasia	Biopsy → insufficient tissue to perform CK7 staining	Slightly positive (±): ductular metaplasia
CD34	Positive (+); more CD34 expression than normal parenchyma, but not diffuse and therefore not suspicious for HCC	Positive (+); especially around the fibrous tissue, but not diffuse and therefore not suspicious for HCC	Strongly positive (++) around the vessels, but not diffuse and therefore not suspicious for HCC	Slightly positive (±): vascularization around fibrous septa is slightly positive, the rest of lesion is negative	Biopsy → insufficient tissue to perform CD34 staining	Biopsy → insufficient
Histological features	Central scar with extensive fibrous tissue visible in the core of the lesion. Only lesion periphery shows no fibrous tissue	No typical central scar, but increased fibrous tissue throughout lesion	Central scar (fibrous tissue)	No central scar, but areas of scar tissue	Cirrhotic intratumor transformation	Large fibrous component
		Inflammatory component in lesion centre	Vascular proliferation (large vessels with thickened vascular walls)	Many fibrous tissue septae	Many fibrous tissue septae	Increased number of bile-ducts compared to surrounding parenchyma
	Cirrhotic intratumor transformation with inflammatory component in lesion centre	Strong proliferation of well-differentiated bile-ducts especially in lesion periphery	Multiple areas of fibrous tissue	Vascular malformation and degeneration	Strong infiltration with lymphocytes	Both well-differentiated and de-differentiated bile-ducts
			Strong infiltration with lymphocytes	Strong infiltration with lymphocytes	Bile-duct proliferation	Considerable infiltration with lymphocytes
	Bile-duct proliferation, especially in lesion periphery	Extensive ductular metaplasia	Bile-duct proliferation	Strong bile-duct proliferation		
Histology conclusion	Typical FNH	Typical FNH	FNH due to vascular malformation with secondary ischemia	FNH due to vascular malformation with secondary ischemia	Typical FNH	Typical FNH
FNH-type (radiology)	Hypo-intense-with-ring type FNH	Hypo-intense-without-ring type FNH	Inhomogeneous hyperintense type FNH	Inhomogeneous hyperintense type FNH	Inhomogeneous hyperintense type FNH	Iso-intense type FNH

In each of these lesions an inflammatory component with lymphocyte infiltration was observed, although the degree of inflammation differed. Second, an increase in bile-ducts compared to the surrounding, normal parenchyma was observed in all six lesions. Using immunohistochemistry, three different types of bile-ducts were observed, i.e.: CK19 positive pre-existent (normal) bile-ducts, CK7 positive ductular proliferation with or without CK19 positivity, and CK7 positive but CK19 negative ductular metaplasia. The relative proportion of pre-existent bile-ducts, ductular proliferation, and ductular metaplasia differed widely between the six lesions. In addition, the localization (lesion centre, lesion periphery, or both) of the three bile-duct types differed between different lesions.

One FNH lesion showed a “hypointense-with-ring” pattern during the hepatobiliary phases (Tumor A). This lesion was slightly positive for CK19, only in the periphery of the lesion, correlating with pre-existent bile-ducts as well as some ductular proliferation (both well-differentiated bile-ducts). The presence of these well-differentiated bile-ducts in the lesion periphery apparently resulted in contrast uptake and excretion given the ring pattern on hepatobiliary phase. Conversely, the core of this lesion consisted of abundant fibrous tissue surrounded by inflammation and CK7 positive bile-duct metaplasia, resulting in a hypointense appearance of the lesion centre. Also, some CK7 positive bile-duct proliferation was observed mainly toward the periphery of the lesion, and only sparsely in the lesion centre (Fig. 4). Interestingly, despite the presence of some bile-duct proliferation surrounding the fibrous tissue, contrast uptake was absent in that area (given the hypo-intense aspect of the lesions centre on hepatobiliary phase), suggesting that these bile-ducts were not functional. Seemingly, in this lesion the capacity of contrast uptake and excretion was impaired in areas lacking pre-existent bile-ducts but with bile-duct metaplasia, CK7 positive bile-duct proliferation, inflammation and fibrosis. In addition, an upregulation of the angiogenesis marker CD34 was observed in the core of these ring-enhancing lesions, indicating vascular proliferation. To our knowledge, the effect of vascular proliferation on Gadoxetic acid uptake has not yet been established.

Another resected FNH (Tumor B) also presented with a hypointense hepatobiliary pattern, but without an enhancing ring (Fig. 6). Histologically, this lesion was characterized by foci of fibrosis throughout the lesion, but without a central scar. There were no pre-existent bile-ducts in this lesion. CK7 staining was diffusely positive mainly as a result of extensive ductular metaplasia, and in the periphery of the lesion due to ductular proliferation. This ductular proliferation was only partially and weakly CK19 positive. In addition, an upregulation of CD34 was observed throughout the lesion, indicating vascular proliferation. Interestingly, despite the presence of ductular proliferation (which are generally well-differentiated bile-ducts); this lesion showed impaired contrast uptake compared to the surrounding liver parenchyma, suggesting that these proliferative ducts were not fully functional. Similar to Tumor A, this lesion therefore shows that the capacity of contrast uptake and excretion is impaired in areas with bile-duct metaplasia and inflammation, as well as in areas with CK7 positive ductular proliferation but no pre-existent bile-ducts.

Two other resected FNH lesions showed an inhomogeneous hepatobiliary enhancement pattern (Tumors C and D). These lesions were histologically characterized by multiple necrotic areas, judged to be induced by vascular malformations (CD34 positivity), and areas with cirrhotic transformation due to bile congestion (Fig. 3). Furthermore, diffuse foci of fibrous septae were observed in both lesions. In Tumor C, the areas of necrosis and cirrhosis were surrounded by well-differentiated pre-existent bile-ducts and ductular proliferation (CK19 positive areas). CK7 staining was strongly positive for ductular metaplasia, not for ductular proliferation. In Tumor D, CK19 staining was only marginally positive for some pre-existent bile-ducts; no CK19 positive ductular proliferation was observed. On the other hand, this lesion was strongly positive for CK7 positive ductular proliferation throughout the lesion, without any signs of metaplasia. Thus, in contrast to Tumor A and B, these tumors had a more heterogeneous histological appearance. Furthermore, Tumor A and B showed a single predominant hypo-intense core with (A) or without (B) a thin rim of peripheral enhancement, while Tumor C and D showed multiple areas of hypo-intensity due to diminished contrast uptake and excretion alternated with multiple areas of hyperintensity consistent with the presence of well-differentiated (pre-existent) bile-ducts.

In Tumor E and F histology of 18G biopsies was available, therefore limiting the possibility to give details about the proportion or localization of the observed histological features. In Tumor E a similar inhomogeneously hyperintense pattern was observed on hepatobiliary phase as in Tumor C and D. CK7 and CK19 staining were not performed due to limited available tissue. However, hematoxylin and eosin (HE) staining did show strong bile-duct proliferation, which might explain the hyperintense appearance of the lesion. Furthermore, extensive fibrosis and cirrhosis was observed together with a strong lymphocytic infiltration. These areas probably correlate to the areas of diminished contrast uptake. Tumor F showed an iso-intense pattern during the hepatobiliary phases, indicating a similar amount of contrast uptake as the surrounding parenchyma. Histology of Tumor F revealed clear CK19 positive bile-ducts consistent with ductular proliferation and pre-existent bile-ducts. Furthermore, CK7 staining was positive as a result of ductular metaplasia. Finally, hyperplasia of the vessel intima was observed as well as fibrotic areas probably induced by necrosis of the hepatocytes. Contrast uptake is likely to be diminished in these areas. Despite the presence of pre-existent bile-ducts, this lesion showed an iso-intense pattern (similar uptake as surrounding (parenchyma) instead of a hyperintense pattern. Presumably because pre-existent bile-ducts were surrounded by areas of ductular metaplasia and fibrosis.

## Discussion

This study shows that hepatobiliary enhancement patterns of FNH’s may present as one of four patterns: homogeneous hyperintense, inhomogeneous hyperintense, iso-intense, and hypo-intense with or without peripheral enhancement. Histological evaluation revealed differences in number, type, and localization of bile-ducts between individual FNH lesions as well as differences in inflammatory component, extent of fibrous tissue and the presence of vascular proliferation. Comparison of histology with imaging features suggests that the observed enhancement patterns can largely be explained by differences in histological features of these lesions.

Previously, Zech et al. [17] described three hepatobiliary enhancement patterns in FNH (homogeneously hyperintense, inhomogeously hyperintense, and peripheral enhancement), all with a hyperintense nature. In addition, Zech et al. reported that no enhancement occurred during the hepatobiliary phase in 10–12 % of FNH’s. In our study, we observed similar enhancement patterns, although only one out of 26 included lesions was homogeneously hypo-intense during the hepatobiliary phase. Possibly in the study of Zech, lesions with a hypointense-with-ring pattern were described as non-enhancing lesions, interpreting the thin peripheral enhancing rim as part of the surrounding liver parenchyma, resulting in a higher percentage of “non-enhancing” FNH.

In this study, we analyzed the histological features of FNH’s in order to better understand the mechanisms of Gadoxetic acid uptake and excretion in relation to the observed enhancement patterns. A better understanding of enhancement patterns and mechanisms might be beneficiary for lesion characterization, which is important as the prevalence of FNH is high. In this study, FNH was diagnosed in the majority (34/62; i.e., 55 %) of patients who were referred for characterization of an incidental finding in the liver.

Previously it has been shown that FNH’s can have various degrees of inflammation, which is induced by either a response to ischemic injury secondary to vascular malformation or by cholangitis secondary to bile obstruction [24]. Our data support these results as inflammation was detected in all six histologically evaluated lesions, although the degree of inflammation differed strongly between the lesions.

So far, it is accepted that Gadoxetic acid uptake is only possible in functioning hepatocytes and that functioning bile-ducts are necessary for excretion in the bile-system. Therefore, in our histological evaluation special emphasis was put on evaluation of bile-ducts.

Owing to increased intraparenchymal pressure, ductular metaplasia of hepatocytes and proliferation of bile-ducts can be induced [7, 8, 25]. These proliferative bile-ducts can be well-differentiated with preserved function, and therefore, Gadoxetic uptake and accumulation can be expected. In FNH, the increased pressure might be secondary to vascular malformation resulting in increased blood-flow to a specific region compared to the surrounding tissue, or as a result of ischemia [6]. In our study, we observed an increase in well-differentiated bile-ducts (either pre-existent bile-ducts, or well-differentiated proliferative bile-ducts) compared to the surrounding parenchyma in all lesions, although the number of well-differentiated bile-ducts differed between lesions. The increased number of well-differentiated bile-ducts, especially of pre-existent bile-ducts, seemed to correlate with increased contrast accumulation, thereby explaining the hyperintense appearance of FNH’s.

Apart from areas with well-differentiated bile-ducts, areas of hepatocytic bile-duct metaplasia were observed. Bile-duct metaplasia can evolve into proliferation of bile-ducts with diminished functionality and inadequate linkage to the bile canaliculi. Bile-duct metaplasia is initiated by chronic inflammation, cholangitis, or bile flow obstruction and all of these components can to a lesser or greater extent be present in FNH [25]. We observed less contrast uptake in the areas with ductular metaplasia and in some areas with ductular proliferation, which might be explained by the diminished functionality of these bile canaliculi, resulting in locally diminished excretion of Gadoxetic acid.

We also observed differences in the distribution of the different bile-duct types throughout the lesions (lesion centre vs. lesion periphery) and this distribution differed between the hypo-intense-with-ring FNH’s, the hypo-intense FNH’s and the inhomogeneously hyperintense FNH’s. In the ring-type FNH we observed fibrous tissue in the lesion centre surrounded by some inflammation and vascular proliferation with ductular metaplasia, while the lesion periphery consisted mainly of well-differentiated pre-existent bile-ducts without signs of metaplasia, fibrous tissue, or inflammation. Although the hypo-intense FNH (lesion B) demonstrated pronounced ductular proliferation in the lesion periphery, no ring-phenomenon was observed in the hepatobiliary phase. This was probably because the periphery also consisted of ductular metaplasia, and pre-existent bile-ducts were lacking. In both inhomogeneously hyperintense FNH’s with resected specimens available, we observed more inflammation and lymphocyte infiltration, with multiple areas of fibrous tissue throughout the lesions that were accompanied by areas of vascular malformation. Interestingly, bile-duct metaplasia was only observed in one of these lesions (Tumor C). Both lesions also contained large areas with ductular proliferation (CK19 positive in Tumor C, CK7 positive in tumor D) containing well-differentiated bile-ducts, while pre-existent bile-ducts were predominantly observed in Tumor C. These well-differentiated bile-ducts might explain the hyperintense appearance of these lesions on hepatobiliary phase. Bile-duct localization could not be assessed accurately in both lesions with histology obtained by means of 18G core-biopsy.

There are several limitations to our study. Histology was only available in six lesions, whilst the standard of reference for the other 20 FNH lesions consisted of typical findings on pre-contrast and early dynamic sequences. However, the stringent criteria used to non-invasively identify these FNH lesions are generally accepted as pathognomonic for FNH, obviating the need for further imaging or follow-up. We did not perform quantification of the various components of the hepatobiliary phase enhancement patterns, reasoning that visual assessment is the cornerstone for pattern recognition and quantification of the observed phenomena would not serve a practical purpose. The inclusion of only FNH lesions that were either proven on pathology or those demonstrating the complete set of pathognomonic MR imaging features on pre-contrast and early dynamic phases, leaves the possibility that other FNH lesions, with one or more atypical features on pre-contrast or early dynamic MR imaging, might show different hepatobiliary enhancement patterns. Therefore, we performed a post hoc analysis on the 14 patients who had received a clinical diagnosis of having at least one FNH lesion but who, according to the study protocol, were excluded from further analysis because of one or more atypical MR imaging features of the observed FNH lesions. A total of 20 lesions clinically characterized as FNH were detected in these 14 patients. All 20 lesions could be categorized into one of the four described hepatobiliary enhancement patterns (homogeneously hyperintense *n* = 11, inhomogeneously hyperintense *n* = 3, iso-intense *n* = 5, ring-type *n* = 1) and no additional enhancement patterns were seen. All 14 patients received a minimum of 1 year follow-up with MRI, demonstrating no growth or change of the observed morphological features, consistent with the clinical diagnosis of FNH. Finally, the design of the study leaves the possibility that lesions with other pathology than FNH might also show one of the observed hepatobiliary enhancement patterns. Although we cannot rule out this possibility, we have not observed the described hepatobiliary enhancement patterns in other pathology than FNH.

In conclusion, FNH lesions can be categorized into one of four hepatobiliary enhancement patterns on Gadoxetic-acid-enhanced MRI. These various enhancement patterns appear to be associated with histological differences in number and type of bile-ducts, and with varying the presence and distribution of fibrous tissue, inflammation, and vascularization.
